# Morphological and Functional Alterations in the CA1 Pyramidal Neurons of the Rat Hippocampus in the Chronic Phase of the Lithium–Pilocarpine Model of Epilepsy

**DOI:** 10.3390/ijms25147568

**Published:** 2024-07-10

**Authors:** Tatyana Y. Postnikova, Georgy P. Diespirov, Sergey L. Malkin, Alexander S. Chernyshev, Elizaveta N. Vylekzhanina, Aleksey V. Zaitsev

**Affiliations:** 1 Sechenov Institute of Evolutionary Physiology and Biochemistry of RAS, Saint Petersburg 194223, Russia; tapost2@mail.ru (T.Y.P.); diespirov.gp@yandex.ru (G.P.D.); adresatt@gmail.com (S.L.M.); elizaveta.vyl@gmail.com (E.N.V.); 2 Ioffe Institute, Saint Petersburg 194021, Russia; tchernysheff@mail.ru

**Keywords:** temporal lobe epilepsy, 4-aminopyridine model, pyramidal neuron, action potential, ictal discharge, hippocampus, entorhinal cortex

## Abstract

Epilepsy is known to cause alterations in neural networks. However, many details of these changes remain poorly understood. The objective of this study was to investigate changes in the properties of hippocampal CA1 pyramidal neurons and their synaptic inputs in a rat lithium–pilocarpine model of epilepsy. In the chronic phase of the model, we found a marked loss of pyramidal neurons in the CA1 area. However, the membrane properties of the neurons remained essentially unaltered. The results of the electrophysiological and morphological studies indicate that the direct pathway from the entorhinal cortex to CA1 neurons is reinforced in epileptic animals, whereas the inputs to them from CA3 are either unaltered or even diminished. In particular, the dendritic spine density in the *str. lacunosum moleculare*, where the direct pathway from the entorhinal cortex terminates, was found to be 2.5 times higher in epileptic rats than in control rats. Furthermore, the summation of responses upon stimulation of the temporoammonic pathway was enhanced by approximately twofold in epileptic rats. This enhancement is believed to be a significant contributing factor to the heightened epileptic activity observed in the entorhinal cortex of epileptic rats using an ex vivo 4-aminopyridine model.

## 1. Introduction

Epilepsy is a common neurological disorder affecting approximately 50 million people worldwide [1]. This disorder is characterized by recurrent spontaneous seizures resulting from hyperexcitability and hypersynchrony of brain neurons [2]. Despite the availability of numerous antiseizure drugs, approximately 30% of patients with epilepsy, specifically temporal lobe epilepsy (TLE), continue to experience seizures [3]. Consequently, the investigation of the mechanisms underlying the generation of ictal activity and the precise functions of distinct brain divisions in this process represents a pivotal objective within the field of experimental neurobiology.

In many cases of temporal lobe epilepsy, impaired interactions between the hippocampus and entorhinal cortex play an important role in the generation of ictal activity. The synaptic connections between the entorhinal cortex and the hippocampus can be described in a simplified manner as follows: there are two parallel excitatory pathways, namely the trisynaptic pathway (entorhinal cortex, layer 2 → dentate gyrus → CA3 → CA1 → entorhinal cortex, layer 5/6) and the monosynaptic direct pathway (entorhinal cortex, layer 3 → CA1) [4]. The direct and trisynaptic pathways from the entorhinal cortex terminate at distinct locations within the dendrites of CA1 pyramidal neurons, as they traverse distinct layers of the hippocampus. Schaffer collaterals, as part of the trisynaptic pathway, are located in the radial layer, while the monosynaptic pathway is situated within the *stratum lacunosum moleculare* [4]. Such an organization of connections in the normal state allows CA1 neurons to match and integrate information from the two pathways [5]. However, in epilepsy, this is likely the reason for the excitotoxicity and increased vulnerability of this area. For instance, temporal lobe epilepsy (TLE) is frequently accompanied by hippocampal sclerosis, which has been demonstrated in MRI, surgical, and post-mortem studies [6,7] and in animal models [8,9]. Such disorders are frequently characterized by cell loss and gliosis in the CA1 and CA3 subfields of the hippocampus, hilus [10,11]. The death of some hippocampal neurons and the reorganization of connections within the hippocampus and other brain structures may be one of the reasons for the maintenance of pathological activity in the brain.

The present study focused on the properties of hippocampal CA1 neurons using a lithium–pilocarpine model of epilepsy in young rats. This model is capable of reproducing the same types of lesions observed in patients with temporal lobe epilepsy. These include significant neuronal death in the CA3 and CA1 regions of the hippocampus, gliosis, proliferation of granular neurons in the dentate gyrus, and mossy fiber sprouting. Furthermore, the phenomenon of pharmacoresistance to antiseizure drugs is frequently observed in this model, which mirrors the situation observed in human temporal lobe epilepsy [12]. Previously, we have demonstrated a reduction in synaptic neurotransmission through Schaffer’s collaterals between the CA3 and CA1 regions of the hippocampus in the lithium–pilocarpine model. Furthermore, we observed a high background activation of the glutamatergic system and an increase in the frequency of spontaneous events on CA1 pyramidal neurons [13]. It is hypothesized that the death of a portion of hippocampal neurons may result in aberrant sprouting, which in turn may lead to altered afferent excitatory connectivity in the CA1 subfield. Therefore, the present study aimed to characterize the electrophysiological membrane properties of CA1 pyramidal neurons, their synaptic inputs from CA3 and the entorhinal cortex, as well as features of epileptiform activity in brain slices obtained from control and lithium–pilocarpine-treated epileptic rats.

## 2. Results

### 2.1. In Epileptic Rats, There Is a Notable Loss of Pyramidal Neurons in the CA1 Region of the Hippocampus, Yet the Membrane Properties of the Neurons Remain Largely Unaltered

**Figure 1:**
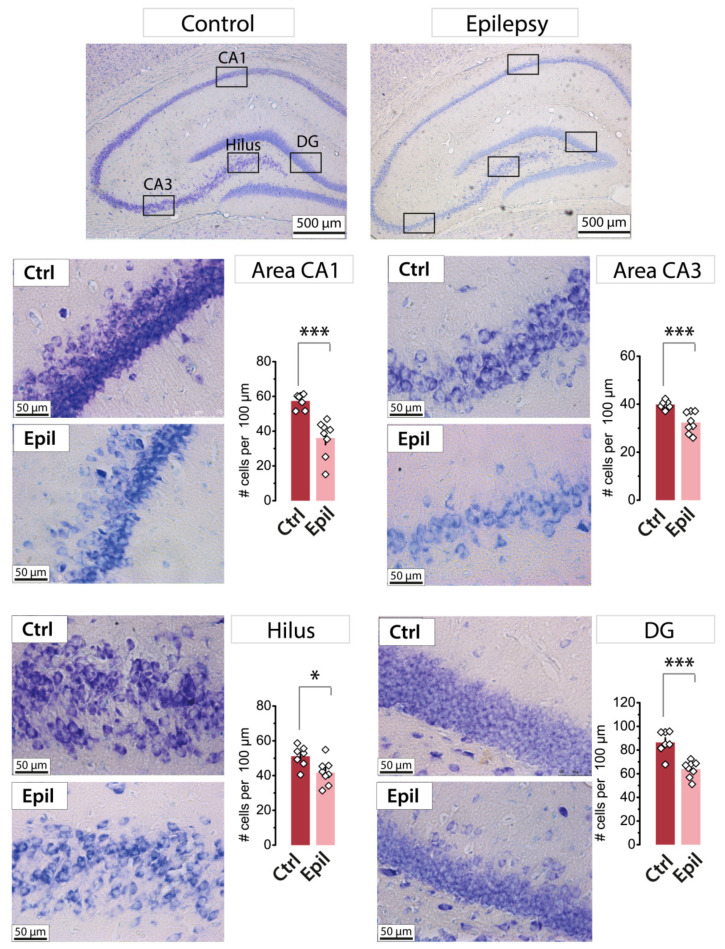
Nissl staining of neurons in the hippocampus in control (*n* = 7) and epileptic (*n* = 8) rats. The diagrams show the average number of Nissl-stained neurons per 100 µm of the cell layer. The dots show the individual values for each rat. Asterisks denote significant differences between groups based on Student’s *t*-test: * *p* < 0.05; *** *p* < 0.001.

It has been demonstrated that the hippocampus is one of the most severely damaged brain structures in the chronic phase of the lithium–pilocarpine model [14]. A distinctive feature of our experimental model is that pilocarpine was administered to rats at the age of three weeks. One month following the induction of status epilepticus by pilocarpine, a quantitative analysis of neuronal loss was conducted in different regions of the hippocampus (Figure 1). This period aligns with the chronic stage of the model, during which time spontaneous recurrent seizures were observed in the majority of experimental animals included in the study.

A statistically significant decrease in the number of neurons was observed in all analyzed areas. The CA1 area exhibited the greatest neuronal death, with 37% of cells lost, followed by the dentate gyrus (26%), CA3 area (19%), and hilus (18%). These findings indicate that the CA1 area is one of the most vulnerable regions of the hippocampus in the lithium–pilocarpine model of epilepsy.

A significant loss of neurons in the CA1 region may result in alterations to their biophysical characteristics. Given that the number of cells has decreased, it can be postulated that the remaining neurons assume the functions of the dead ones. As a result, it may be expected that the size of the surviving neurons may increase due to an expansion in the total length of dendrites and axons. This would consequently result in alterations to the passive membrane properties, specifically a reduction in input resistance and an elongation of τ. We therefore proceeded to record and analyze the membrane properties of pyramidal neurons via whole-cell patch clamp recording.

**Figure 2:**
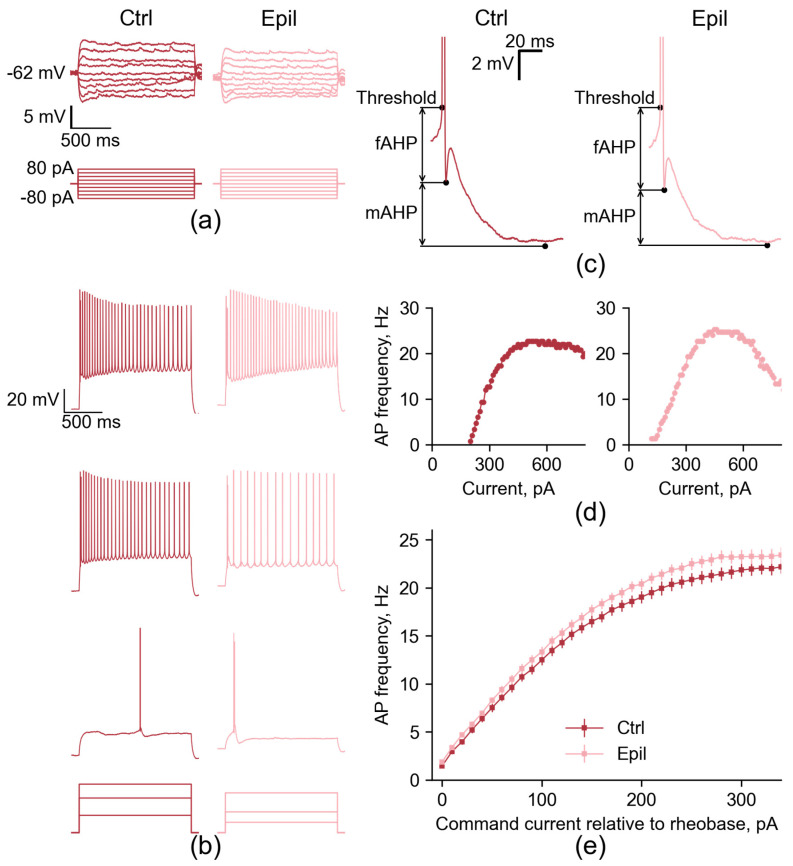
Firing patterns of CA1 pyramidal neurons in control (Ctrl) and epileptic (Epil) rats. (**a**) Representative examples of the membrane responses to the steps of hyperpolarizing and subthreshold depolarizing current in CA1 neurons from control and epileptic animals showing that the membrane input resistance and τ are unaltered. (**b**) Representative examples of the membrane responses of CA1 neurons to the depolarizing steps of the rheobase current (bottom), 2 x rheobase current (middle), and current sufficient to elicit the depolarizing block (top). (**c**) Representative examples of the fast and medium AHP phases of the APs in CA1 neurons. (**d**) The current–frequency curves for the same neurons shown in (**b**). (**e**) Averaged current–frequency curves of CA1 neurons.

However, the membrane input resistance and τ were unaltered (Figure 2a, Table 1), thereby refuting our hypothesis. It is also notable that the resting membrane potential of neurons from the epileptic hippocampus did not differ from that of controls.

Next, we compared the firing patterns of the CA1 neurons of the epileptic rats with the age-matched control. Representative examples of the action potential (AP) trains generated in response to the suprathreshold depolarizing current steps in CA1 neurons are shown in Figure 2b.

**Figure 3:**
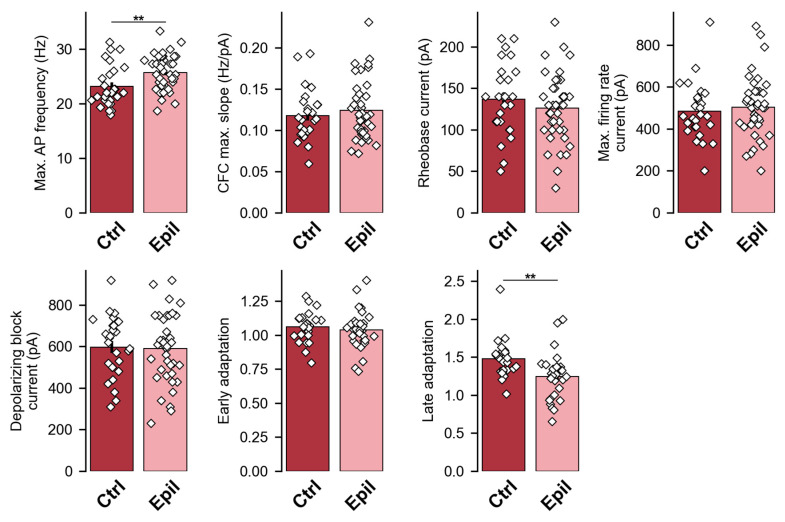
Firing properties in CA1 pyramidal cells from control (Ctrl) and epileptic (Epil) rats. The dots show the individual values for each neuron. Asterisks denote significant differences between groups based on Student’s *t*-test: ** *p* < 0.01.

The overall response patterns were very similar between groups; however, CA1 neurons from epileptic rats showed an increased maximum AP firing rate compared to controls (control: 23.2 ± 0.7 Hz; epileptic: 25.7 ± 0.5 Hz; *p* < 0.01; Figure 2 and Figure 3). This change was accompanied by an 18% reduction in late frequency adaptation of APs (control: 1.48 ± 0.05; epileptic: 1.25 ± 0.05; *p* < 0.01), but not in early adaptation (control: 1.06 ± 0.02; epileptic: 1.04 ± 0.03; *p* = 0.5). However, all other parameters of the firing pattern in the CA1 neurons of the epileptic rats were similar to the control. The maximum slope of the current–frequency curve, which characterizes the rate of increase in AP frequency with increasing depolarizing current amplitude, was 0.12 ± 0.01 in control and 0.12 ± 0.01 in epileptic rats (*p* = 0.47). The current amplitudes sufficient to evoke the first APs (control: 137 ± 8 pA; epileptic: 126 ± 7 pA; *p* = 0.32), the maximum frequency of the APs (control: 484 ± 26 pA; epileptic: 503 ± 23 pA; *p* = 0.6), and the depolarizing block (control: 597 ± 28 pA; epileptic: 590 ± 26 pA; *p* = 0.86) were also similar in these two groups of rats (Figure 3). Consequently, the altered excitability parameters observed in CA1 neurons of epileptic rats are predominantly associated with conditions of extreme excitation, where these neurons are capable of sustaining slightly elevated firing rates before entering depolarizing block.

Subsequently, the properties of individual APs in CA1 neurons were evaluated. The following parameters were measured: AP threshold, amplitude, half-width, and rise time from 10 to 90% of the amplitude of the AP; fast and medium afterhyperpolarization (AHP) amplitude and timing; as well as the amplitude of afterdepolarization (ADP). These parameters are largely defined by the kinetics of different sodium and potassium channels [15]. No significant alterations were observed in the action potential properties of CA1 pyramidal cells in the chronic phase of the lithium–pilocarpine model (Table 2).

### 2.2. In Epileptic Rats, There Is a Change in the Ratio of Inputs from the Entorhinal Cortex and the CA3 Region of the Hippocampus to CA1 Pyramidal Neurons

Given that no significant alterations were observed in the membrane properties of pyramidal neurons, while the functionality of neuronal networks in the epileptic hippocampus was found to be disrupted [13], it can be postulated that synaptic connections may be disrupted. Previously, we have demonstrated a reduction in synaptic neurotransmission through Schaffer’s collaterals between the CA3 and CA1 regions of the hippocampus in the lithium–pilocarpine model. In addition, we observed a high background activation of the glutamatergic system and an increase in the frequency of spontaneous events on CA1 pyramidal neurons [13]. It is possible that the observed changes are caused by an increase in neurotransmission through the direct pathway from layer 3 of the entorhinal cortex to the CA1 region of the hippocampus [4,16]. This hypothesis is supported by evidence from previous studies [17]. Consequently, we conducted a study to examine the properties of synaptic neurotransmission between the CA3 and CA1 regions and between the entorhinal cortex and the CA1 region of the hippocampus in control rats and rats in the chronic phase of the lithium–pilocarpine model of temporal lobe epilepsy. Whole-cell patch clamp recordings were conducted on CA1 pyramidal neurons, with electrical extracellular stimulation applied to two distinct pathways to elicit evoked excitatory postsynaptic currents (eEPSCs).

**Figure 4:**
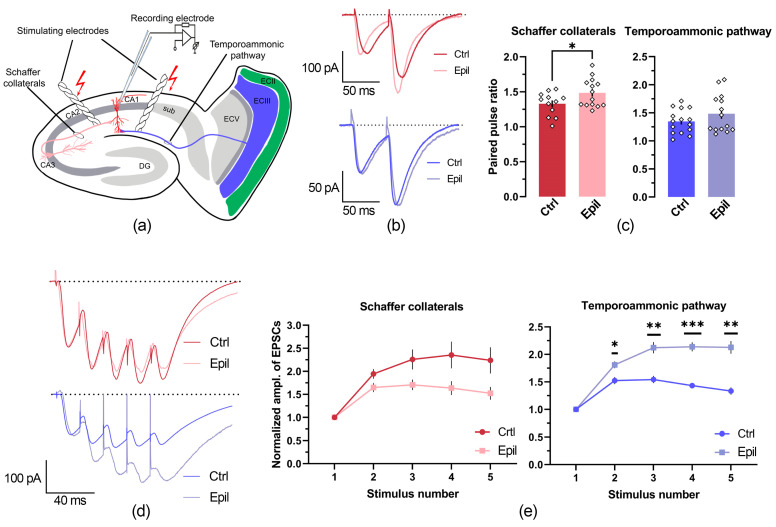
The inputs from the entorhinal cortex and the CA3 region of the hippocampus to CA1 pyramidal neurons are altered in epileptic rats. (**a**) Schematic representation of the location of the electrodes used for the stimulation of Schaffer’s collaterals and the temporoammonic pathways. (**b**,**d**) Representative examples of recordings of two-pulse (**b**) and train (**d**) evoked excitatory postsynaptic currents (eEPSCs) of Shaffer collaterals (red) and temporoammonic pathway (blue) in control (ctrl) and epileptic (epil) rats. (**c**) Bar graphs illustrating the paired-pulse ratios in the various groups. The dots show the individual values for each neuron. * *p* < 0.05, Student’s *t*-test. (**e**) Normalized amplitude of eEPSCs obtained during short-train stimulation. A repeated measures ANOVA was conducted, followed by the Šidák post hoc test; * *p* < 0.05, ** *p* < 0.01, and *** *p* < 0.001.

First, we compared the amplitude ratios obtained during paired-pulse stimulations of Schaffer’s collaterals (CA3 → CA1) and the temporoammonic pathway (entorhinal cortex → CA1). The interstimulus interval was 50 ms in both cases (Figure 4a–c).

In epileptic rats, the paired-pulse ratio during stimulation of Schaffer collaterals was found to be greater than in control animals (t = 2.13, *p* < 0.05). In contrast, this ratio did not change in the case of temporoammonic pathway stimulation. This finding indicates that in the epileptic group, the probability of mediator release from the postsynaptic terminals of CA3 neurons is reduced, whereas this probability remains unchanged in the terminals of entorhinal neurons.

Subsequently, we conducted an analysis of the summation of EPSCs evoked by a short-train of five stimuli with a frequency of 50 Hz (Figure 4d,e). It was observed that in epileptic rats, the summation of responses evoked by stimulation of the temporoammonic pathway was significantly higher (F_1, 10_ = 54.6, *p* < 0.001), whereas the summation of responses evoked by stimulation of the Schaffer’s collaterals decreased (F_1, 14_ = 5.28, *p* < 0.05).

As Schaffer’s collaterals terminate at apical dendrites, which are situated in the stratum radiatum, and the temporoammonic pathway terminates in the distal portion of the apical dendrites of CA1, which are located within the stratum lacunosum moleculare [4], it was deemed pertinent to ascertain whether there existed any structural correlates of the observed functional alterations.

**Figure 5:**
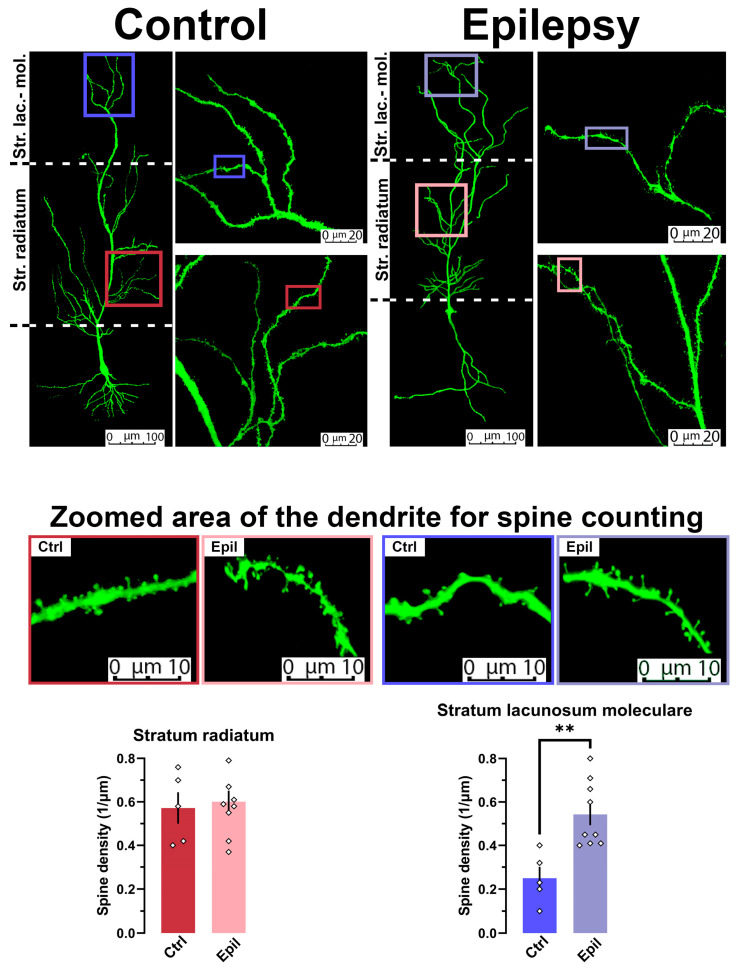
In epileptic rats, spine density is observed to increase on apical dendrites of pyramidal neurons in stratum lacunosum moleculare. The images above illustrate examples of biocytin-filled and confocal reconstructed pyramidal neurons in control and epileptic rats at different magnifications. The bottom bar diagrams illustrate the density of spines on dendrites of CA1 pyramidal neurons, with the data presented in different layers. The dots show the individual values for each neuron. ** *p* < 0.01, Student’s *t*-test.

The filling of neurons with biocytin during electrophysiological recordings permitted the morphological reconstruction of these cells (Figure 5). The density of dendritic spines in the stratum radiatum and stratum lacunosum moleculare of control and epileptic rats was analyzed. The results demonstrated that the spine density in the stratum lacunosum moleculare of chronic TLE rats was more than 2.5 times higher than that of control rats (t = 3.74, *p* < 0.01). However, no differences were observed in the stratum radiatum.

Consequently, both electrophysiological and morphological data indicate that the direct pathway from the entorhinal cortex to CA1 neurons is reinforced, whereas the inputs to them from CA3 are either unaltered or even diminished.

### 2.3. Epileptiform Activity Induced by 4-Aminopyridine in Hippocampus–Entorhinal Cortex Slices Differs between Control and Epileptic Rats

**Figure 6:**
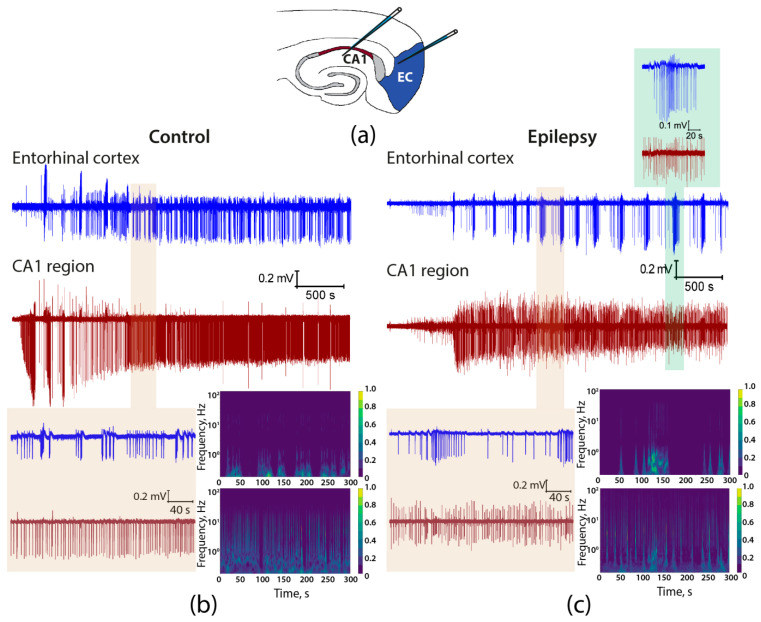
Epileptiform activity induced by 4-aminopyridine in hippocampus–entorhinal cortex slices. (**a**) The drawing shows the position of the electrodes in the hippocampus and entorhinal cortex. Simultaneous LFP recordings in brain slices from control (**b**) and epileptic (**c**) rats showing the development of epileptiform activity after the application of a proepileptic solution. Expanded views of a representative period of epileptiform activity are displayed on a light brown background, with corresponding spectrograms shown on the right-hand side. Low-amplitude LFP changes correlating with ictal discharge are observed in the hippocampus of epileptic animals during ictal discharge in the entorhinal cortex (inset, light green background).

The subsequent step was to ascertain whether there were differences in the generation of epileptiform activity in brain slices of control and epileptic rats. To this end, a 4-aminopyridine ex vivo model was employed, and epileptiform activity was analyzed in brain slices containing the hippocampus and entorhinal cortex. Simultaneous recordings of local field potentials (LFPs) were obtained from the radial layer of the CA1 area of the hippocampus and the deep layers of the entorhinal cortex (Figure 6). Following the application of an epileptogenic solution, epileptiform activity rapidly developed with approximately the same delay in both groups in hippocampus (control: 99.0 ± 15.4 s, *n* = 12; vs. epileptic: 92.6 ± 19.2 s, *n* = 9, *t* = 0.27, *p* = 0.79) and the entorhinal cortex (control: 183.7 ± 26.7 s, *n* = 12; vs. epileptic: 244.4 ± 51.4 s, *n* = 9, *t* = 1.13, *p* = 0.27).

**Figure A1:**
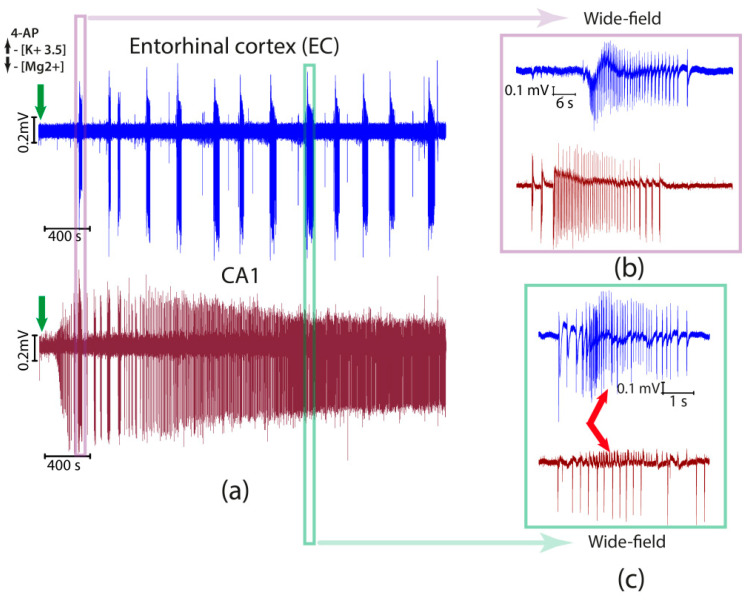
Epileptiform activity induced by 4-aminopyridine in hippocampal–entorhinal cortex slices. (**a**) Simultaneous LFP recordings in brain slices from epileptic rats showing the development of ep-ileptiform activity after application of a proepileptic solution. Expanded views of an initial (**b**) and a steady (**c**) period of epileptiform activity are shown in the frames.

Visual analysis of the recordings revealed significant differences between the epileptic and control animals (Figure 6). For example, in the hippocampus, interictal discharges were more frequent in control rats than in epileptic animals. In most control animals, interictal activity was predominant in the entorhinal cortex, and when ictal discharges occurred, they were of relatively short duration. In the epileptic animals, ictal discharges predominated in the entorhinal cortex (Figure A1) and lasted longer than in the control group. Furthermore, these ictal discharges partially propagated from the entorhinal cortex to the hippocampus, but the amplitude of the “ictal” LFP in the hippocampus was relatively small (Figure 6, inset). The propagation of ictal discharges from the entorhinal cortex to the hippocampus was not observed in control animals.

The use of wavelet transformation in signal analysis enables a clearer visualization of the distinction between the control and epileptic groups. Specifically, the intensity of ictal activity in the entorhinal cortex is greater in epileptic rats. In control animals, the interictal activity is more prominent than in epileptic animals and propagates from the hippocampus to the entorhinal cortex; thus, the interictal discharges in the hippocampus and cortex are more synchronized in control animals than in epileptic animals (Figure 6).

**Figure 7:**
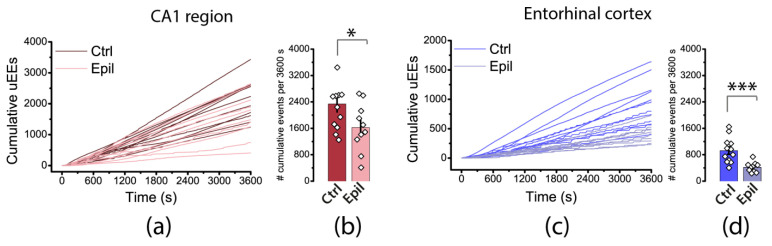
Cumulative plots of unitary epileptiform events (uEEs) in the hippocampus (**a**) and entorhinal cortex (**c**) of control and epileptic rats. The bar graphs on the right-hand side (**b**,**d**) display the average number of uEEs per hour of recording, along with their standard error of measurement. Each point on the graph represents one brain slice. Asterisks indicate significant differences between groups according to Student’s test: * *p* < 0.05; *** *p* < 0.001.

To compare patterns of epileptiform activity quantitatively, we binarized the signal and identified unitary epileptiform events (uEEs) in the hippocampus and entorhinal cortex throughout the recording using the algorithm described in the Methods section. We then created a cumulative plot of the number of uEEs within one hour of the change in the bath solution to a proepileptic one for each slice. The algorithm identifies ictal discharges as sets of closely spaced uEEs, while interictal discharges usually correspond to a single uEE or a short burst of uEEs. Therefore, on cumulative curves, the steeply rising part corresponds to an ictal discharge (Figure 7). As shown in the figure, these steps are noticeable in the entorhinal cortex curves and practically absent in the hippocampus. The total number of uEEs in the hippocampus (2337 ± 242 vs. 1625 ± 229) and in the cortex (925 ± 111 vs. 419 ± 48) was lower in epileptic rats than in controls. Thus, epileptiform activity in brain slices from rats with epilepsy differs both qualitatively and quantitatively from that in controls.

**Figure 8:**
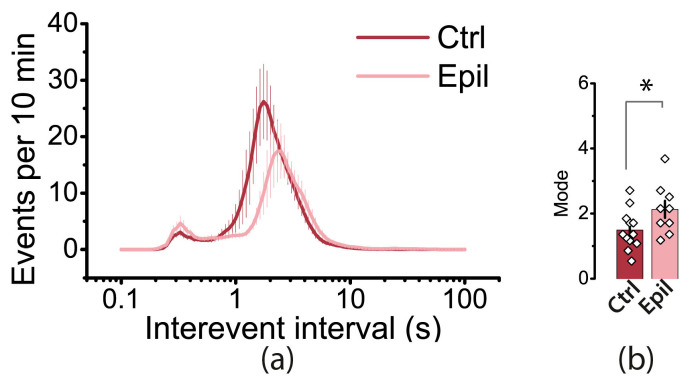
The frequency of uEEs may be reduced due to neurodegeneration in the hippocampus. The interevent intervals (IEIs) distributions in the hippocampi of control and epileptic rats are shown in (**a**). The bar graphs in (**b**) display the averages of the greatest mode of distributions of IEIs in control and epileptic rats. Each point on the graph represents one brain slice. Asterisk indicates significant differences between groups according to Student’s test: * *p* < 0.05.

We then analyzed the distribution of interevent intervals (IEIs) in the hippocampus (Figure 8). In both control and epileptic rats, the distributions had a bimodal shape. The first mode is in the region of 2 s, which roughly corresponds to the frequency of interictal events. In control animals, this mode was shorter (1.5 ± 0.2 s) than that in epileptic rats (2.1 ± 0.3 s, *t* = 2.12, *p* < 0.05). The second mode occurs at approximately 0.3 s, which is more indicative of events that are part of the ictal discharge. It is important to note that while interictal events were more common in both groups, the proportion of events with short IEIs increased in the epilepsy group.

**Figure 9:**
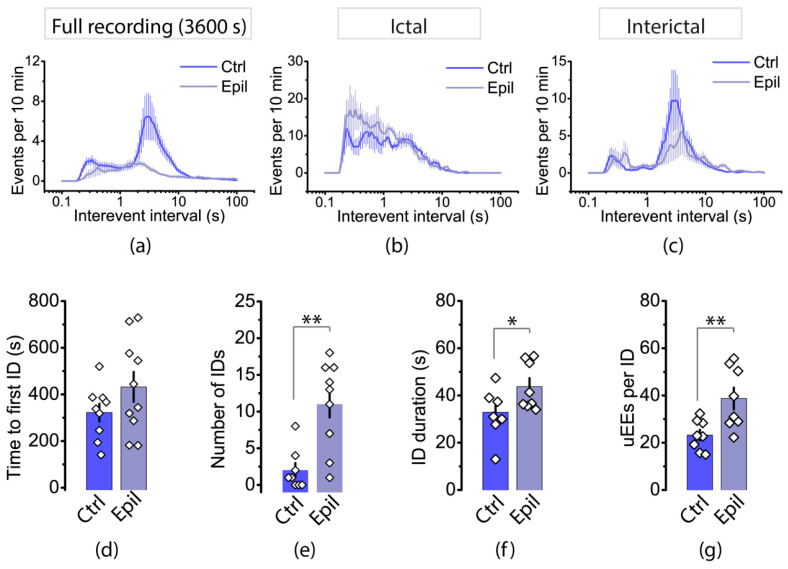
The distribution of IEIs in the entorhinal cortex of control and epileptic rats for 1 h recordings (**a**) and for only ictal (**b**) and interictal (**c**) discharges. The bar graphs display the properties of the ictal discharges, including the latency of the first ictal discharge (**d**), the number of ictal discharges during 1 h recordings (**e**), the duration of ictal discharge (**f**), and the number of uEEs within an ictal discharge (**g**). Each dot on the graph represents one brain slice. Asterisks denote significant differences between groups based on Student’s *t*-test: * *p* < 0.05; ** *p* < 0.01.

The distribution of IEIs in the entorhinal cortex differed significantly between control and epileptic rats (Figure 9a). In control animals, the distribution of IEIs was similar to that observed in the hippocampus, with a predominant mode indicating interictal discharges. However, in epileptic animals, the distribution is different, and the proportion of interictal discharges is lower. To compare the features of epileptiform activity in the cortex of epileptic and control animals more accurately, we isolated areas with ictal and interictal activity on the recording and analyzed the distribution of IEIs (Figure 9b,c). Our findings indicate that ictal discharges in epileptic and control rats occurred with approximately equal latency after the application of the epileptic solution (300–400 s). However, ictal activity in the entorhinal cortex disappears rapidly in control rats. On average, we recorded 2.0 ± 0.9 ictal discharges per hour (*n* = 8). In epileptic rats, ictal activity was significantly greater, with 11.0 ± 2.0 discharges per hour (*n* = 9, *t* = 3.86, *p* < 0.01). In control rats, no ictal activity was recorded in 37.5% of the slices, while in epileptic rats, ictal activity was present in 100% of the slices.

The results showed that ictal discharges were greater in epileptic rats than in control rats (Figure 9f; control: 32.9 ± 3.6 s, *n* = 8; vs. epileptic: 43.8 ± 3.5 s, *n* = 8, *t* = 2.17, *p* < 0.05), consisted of more uEEs (Figure 9g; control: 23 ± 2, *n* = 8; vs. epileptic: 39 ± 5, *n* = 8, *t* = 3.04, *p* < 0.01), and occurred more frequently (Figure 9b). Interictal discharges were observed in control animals approximately once every 2–4 s, whereas in epileptic animals they occurred once every 3–5 s (Figure 9c).

## 3. Discussion

The present study focused on the properties of pyramidal neurons in the hippocampal CA1 region of epileptic rats in the lithium–pilocarpine model. Our findings indicate that in this model of epilepsy, the CA1 region is one of the most vulnerable, with maximal death of hippocampal neurons observed here. However, electrophysiological membrane properties of pyramidal neurons of this area are practically not disturbed. It can be observed that the altered excitability parameters observed in CA1 neurons of epileptic rats are predominantly associated with conditions of extreme excitation, where these neurons are capable of sustaining slightly elevated firing rates before entering the depolarizing block. Concurrently, the synaptic inputs of pyramidal neurons underwent a significant modification. The direct input from the entorhinal cortex increased, accompanied by the appearance of new synaptic contacts. In contrast, the input from the CA3 area remained unchanged or weakened slightly. These changes in synaptic inputs significantly altered the characteristics of epileptiform activity in an ex vivo 4-AP model. In the CA1 region of the hippocampus, interictal discharges were more frequent in control animals than in epileptic rats. In the entorhinal cortex of control rats, interictal activity was predominant, and ictal discharges, when they occurred, were relatively short in duration. In the entorhinal cortex of animals with epilepsy, ictal discharges occur more frequently and last longer than in the control group.

Temporal lobe epilepsy is frequently accompanied by damage to the CA1 region of the hippocampus, including neuronal loss and gliosis. This has been demonstrated in both patients and experimental studies [18,19,20,21]. Our data obtained in this work are fully consistent with this. Previous studies have demonstrated that the expression of many ion channels, including transient receptor potential vanilloid 1 (TRPV1) [22], hyperpolarization-activated cyclic nucleotide-gated channels (HCNs) [23,24,25], and small-conductance Ca^2+^-activated K^+^ (SK) channels [26] can be altered during epileptogenesis. Furthermore, specific changes in sodium channel expression [27,28,29] and A-type voltage-gated potassium (Kv) channels [30] were also identified in CA1 neurons. We hypothesized that changes in ion channel expression would be evident in the assessment of biophysical membrane properties of pyramidal neurons, as these properties depend on different types of ion channels. For example, AP threshold, amplitude, and rise time from 10 to 90% of the amplitude of the AP depend mostly on the voltage-gated sodium channels, AP amplitude, AP half-width and fast afterhyperpolarization (AHP) amplitude, which are mostly defined by the kinetics of the potassium channels, as well as the amplitude and timing of medium AHP, which is mediated by the big-conductance (BK) K^+^ channels, and ADP amplitude, which is also defined by the K_V_ channel activity [15,31,32]. Nevertheless, no discernible distinctions were observed between the control and epileptic animals in terms of the biophysical membrane characteristics of pyramidal neurons.

One of the most interesting results of the present study is the increase in spine density on the distal portions of apical dendrites of CA1 pyramidal neurons. Dendritic spines that represent the morphological sites of the majority of excitatory synaptic inputs could be critically involved in the pathophysiology of epilepsy [33,34]. Changes in distribution and number of dendritic spines in cortex and hippocampus have been identified both after acute convulsions induced by kainic acid [35], monosodium glutamate [36], and pilocarpine [37], and in models of chronic epilepsy [37,38,39]. Loss of dendritic spines has been observed in most of the above-mentioned studies. Similar alterations in dendritic morphology and spine loss mainly in hippocampal neurons have been reported in human brain tissues from patients with epilepsy [40,41].

A greater number of synaptic contacts was observed in a smaller number of studies. For instance, researchers observed synaptic reorganization in the hippocampus, with excitatory connections between CA1 pyramidal cells increasing following kainate-induced status epilepticus [42]. In patients with TLE, the proximal dendrites of dentate granule cells exhibit a greater spine density where the aberrant collaterals were densely localized [43]. The increase in spine density on distal parts of dendrites of CA1 neurons indicates an increase in excitatory inputs from the entorhinal cortex. This was demonstrated directly by measuring the summation of responses evoked by stimulation of the temporoammonic pathway. The summation of responses was significantly higher in epileptic rats compared to controls. Previous studies have also shown that the temporoammonic pathway, a direct cortical input to hippocampal area CA1, is enhanced in both the lithium–pilocarpine model [17,44] and the kainate model [45] of epilepsy. EC layer 3 interneurons are known to inhibit the temporoammonic pathway [46], but their loss is thought to play an important role in the activation of the temporoammonic pathway and CA1 field [47]. This transformation causes the pathway to become 10 times more effective as an excitatory projection. Thus, our results are consistent with the concept that epilepsy alters neural networks.

At the next stage of our study, we analyzed the features of ictogenesis in control and epileptic rats. The mechanism of transition to the epileptic state is often studied in ex vivo models. Many laboratories use combined hippocampal–entorhinal cortex slices from rodent brains for this purpose [48,49,50,51]. Epileptiform activity is induced by perfusing brain slices with artificial cerebrospinal fluid (ACSF) containing one of several chemoconvulsants, such as the K^+^ channel blocker 4-aminopyridine (4-AP) [52,53], the GABAa receptor antagonists bicuculline [54] or picrotoxin [55], the glutamate receptor agonist kainate [56], or by using a nominally zero Mg^2+^ solution [57]. Multiple studies have demonstrated that neuronal networks in brain slices interact to produce patterns of epileptiform activity that resemble the limbic seizures commonly observed in patients with TLE [50,58,59,60]. Here, a 4-AP ex vivo model of epileptiform activity was used.

At least two types of epileptiform discharges are typically distinguished based on differences in duration, spread, amplitude, initiation site, and response to antiepileptic drugs: interictal discharges and ictal or seizure-like events [51]. Interictal discharges are significantly shorter in duration than ictal discharges. Interictal discharges have a heterogeneous origin. Some are caused primarily by the synchronous activity of inhibitory interneurons, while others are caused by the simultaneous activity of excitatory principal neurons [61]. Interictal discharges can have either a proictogenic or anti-ictogenic effect [62,63]. In the case of a proictogenic effect, they are referred to as preictal discharges [60]. In contrast, interictal discharges originating in the CA3 region of the hippocampus and propagating through the CA1 region to the entorhinal cortex can effectively inhibit the generation of ictal activity in this region [64,65].

Various mechanisms of proictogenic action of interictal events have been described. For instance, hypersynchronization of interneurons in the entorhinal cortex can initiate ictal discharge [61,66,67]. Field potential recordings indicate that the ictal discharge in this case is preceded by an isolated ‘slow’ interictal discharge [63]. In contrast, a specific type of interictal discharge, preictal discharge, has been described in the subiculum of tissues from individuals with TLE. Preictal discharges are dependent on glutamatergic activity rather than the more common mixed depolarizing GABA/glutamatergic processes that underlie most interictal discharges. Preictal discharges precede ictal events in vitro [60]. In both cases, ictal discharges in vitro resembled electrographic limbic seizures. Although the current study did not specifically focus on which interictal discharges might trigger ictal events, it is likely that the ictal discharges occurring in the entorhinal cortex are due to ‘slow’ interictal discharge.

In control animals, ictal discharges in the entorhinal cortex, if any, occur only at the very beginning after the addition of the epileptogenic solution and then are replaced by interictal discharges. In vitro studies have shown that interictal activity generated in the hippocampal subfield CA3 has an anti-ictogenic effect when the hippocampal loop is intact [59,68]. In this case, interictal discharges originating in CA3 spread through CA1 and the subiculum to the entorhinal cortex and then re-enter the hippocampus through the dentate gyrus [59]. Transection of Schaffer collaterals, which connect the hippocampal CA3 and CA1 areas, can restore ictal activity in the entorhinal cortex [49,59]. Moreover, low-frequency electrical or optogenetic stimulation of the CA3 or CA1 regions can successfully suppress ictal activity in the entorhinal cortex [65,69,70]. The anti-ictogenic effect of the CA3 area is a characteristic found in adult animals. In young animals, the entorhinal cortex retains ictal activity even when Schaffer collaterals are preserved [71].

Ictal discharges occur more frequently and persist longer in the entorhinal cortex of epileptic animals, which is consistent with the findings of a previous study on pilocarpine-induced epilepsy in mice [68]. In the pilocarpine model, pyramidal neurons and interneurons, particularly in the CA3 and CA1 regions, are lost [21,72], which was confirmed in the present study. The death of neurons in these areas reduces hippocampal output activity and releases its control on entorhinal cortex network excitability [68]. Additionally, dysregulation of excitatory inputs in the hippocampus has been observed. In favor of this transformation, except for the changes in summation during activation of temporoammonic inputs discussed above, we observed that low-amplitude LFP changes correlated with ictal discharge were observed in the hippocampus of epileptic animals during ictal discharge in the entorhinal cortex. Such a phenomenon was not observed in slices of control animals.

In conclusion, the characteristics of epileptiform activity in brain slices obtained from control and lithium–pilocarpine-treated epileptic rats differed. The increased ictal activity in the entorhinal cortex of epileptic animals was most likely due to morphological and functional abnormalities in the CA1 hippocampus and specific amplification of temporoammonic inputs. Furthermore, our findings lend support to the use of low-frequency stimulation of the hippocampus or subiculum, situated downstream of the hippocampus proper, for the control of seizures in drug-refractory epilepsy from a translational perspective [49].

## 4. Materials and Methods

### 4.1. Animals

Male Wistar rats were used in the present study. Rats were maintained under standard conditions at room temperature with ad libitum access to food and water. The animal experiments were conducted in accordance with the ARRIVE guidelines and were performed in accordance with the EU Directive 2010/63/EU on animal experiments. The experimental protocol was approved by the Ethics Committee of the Sechenov Institute of Evolutionary Physiology and Biochemistry (Protocol No. 1-7/2022, 27 January 2022). Every effort was made to minimize the number of animals used and their suffering.

### 4.2. Lithium–Pilocarpine Model of Temporal Lobe Epilepsy

A detailed description of the model has been provided previously [73]. The procedure involved injecting 21-day-old rats intraperitoneally (i.p.) with 127 mg/kg LiCl. After 24 h, the rats were treated with pilocarpine (30 mg/kg, i.p.). To prevent peripheral effects of pilocarpine, (-)-scopolamine methylbromide (1 mg/kg, i.p.) was administered one hour before pilocarpine. Only rats with stage 4 or higher seizures on the Racine scale [74] lasting at least 90 min were included in the study. The control group received LiCl, scopolamine methylbromide, and saline. To confirm the spontaneous seizures, 11 rats from the experimental group, aged 1.5 months, were selected. A 48 h monitoring period was conducted to observe the animals’ behavior and count the number of spontaneous recurrent seizures. Ten out of eleven rats exhibited an average of 2 to 3 clonic seizures during the monitoring period. The observed seizures were rated on the Racine scale, with an intensity of 3 to 4 points.

### 4.3. Slice Preparation and Electrophysiological Recordings

Electrophysiological experiments were performed 30–35 days after the injection of pi-locarpine. Rats were anesthetized with isoflurane (Laboratorios Karizoo S.A., Barcelona, Spain), decapitated, and their brains quickly removed. Brain slices containing the hippocampus and entorhinal cortex were sectioned using a Microm International HM 650 V vibratome (Microm, Walldorf, Germany) in ice-cold carbogen-aerated ACSF containing 126 mM NaCl, 2.5 mM KCl, 1.25 mM NaH_2_PO_4_, 1 mM MgSO_4_, 2 mM CaCl_2_, 24 mM NaHCO_3_, and 10 mM glucose. The slices were then transferred to oxygenated ACSF and incubated at 35 °C for 1 h before electrophysiological recording. The brain slices were then transferred to a recording chamber and perfused with ACSF at a constant flow rate of 5 mL/min for 15–20 min at 30 °C. Local field potentials (LFPs) were recorded simultaneously from the CA1 stratum radiatum and deep layers of the entorhinal cortex using glass microelectrodes (0.2–1.0 MΩ). LFPs were recorded with a Model 1800 amplifier (A-M Systems, Carlsborg, WA, USA) and digitized with an ADC/DAC NI USB-6211 (National Instruments, Austin, TX, USA). Recording was performed using WinWCP v5.7.8 software (University of Strathclyde, Glasgow, UK).

Whole-cell voltage clamp recordings of EPSCs were performed on pyramidal neurons in the CA1 field of the hippocampus. Neurons were visualized using a Zeiss Axioscop 2 microscope (Zeiss; Oberkochen, Germany) equipped with differential interference contrast optics and a PointGrey Grasshopper3 GS3-U3-23S6M-C video camera (FLIR Integrated Imaging Solutions Inc., Wilsonville, OR, USA). Signals were recorded using a Multiclamp 700B (Molecular Devices, Sunnyvale, CA, USA) patch clamp amplifier and an NI USB-6343 A/D converter (National Instruments, Austin, TX, USA) with WinWCP 5 software (University of Strathclyde, Glasgow, UK). A CsMeSO_4_-based pipette solution was used, containing (in mM): 127 CsMeSO_4_, 10 NaCl, 5 EGTA, 10 HEPES, 6 QX314, 4 ATP-Mg, and 0.3 GTP. The pH was adjusted to 7.25 with CsOH. Patch electrodes (4–5 MΩ) were pulled from borosilicate glass capillaries using a P-1000 micropipette puller (Sutter Instrument, Novato, CA, USA). The voltage clamp holding potential was fixed at -20 mV and the response recording potential was −70 mV.

Synaptic responses at a CA1 pyramidal neuron were elicited by stimulating two different inputs using bipolar stimulating electrodes placed at the Shaffer collaterals and the temporoammonic pathway (Figure 4a). An A365 stimulus isolator (World Precision Instruments, Sarasota, FL, USA) was used for extracellular stimulation. The NMDAR channel blocker AP-5 (50 mM, Sigma-Aldrich, St. Louis, MO, USA) and the GABAa blocker bicuculline (20 mM, Sigma-Aldrich) were applied in the bath.

The independence of the inputs was tested by stimulating the first and second pathways with 50 and 200 ms delay, respectively; if the amplitude of the responses at 50 ms did not exceed the amplitude at 200 ms by more than 15%, the stimulating inputs were considered independent. Next, two stimulation protocols were used: paired-pulse (50 ms interstimulus interval) and short-train (5 pulses at 50 Hz) protocols. Stimulation of Schaffer’s collaterals and the temporoammonic pathway was applied alternately at 10 s intervals.

Passive and active membrane properties were investigated using neuronal responses to current steps (1.5 s duration every 3.5 s, amplitudes from −100 to +600 pA in 10–20 pA increments) as previously described [75]. Signals were recorded with the Heka EPC8 amplifier, digitized at 50 kHz frequency using the LIH 8+8 AD-DA converter and PatchMaster software version 2x92 (all from HEKA Elektronik GmbH, Lambrecht, Germany). The patch electrode was filled with the internal solution containing the following (in mM): 135 potassium gluconate, 5 KCl, 5 NaCl, 5 EGTA, 10 HEPES, 4 ATP-Mg, and 0.3 GTP-Na, 0.5% biocytin, pH 7.25, adjusted with KOH.

The resting membrane potential was measured as the voltage at zero input current. Input resistance was estimated as the slope of the linear regression of the current–voltage relationship for the subthreshold steps. Membrane τ was calculated as a parameter of a single exponential function fitted to the onset of the voltage response to a hyperpolarizing current step.

APs generated at the rheobase current were selected for analysis. The rheobase current was determined as the minimum current sufficient to induce AP generation. The voltage threshold was determined as the point at which the first derivative of the voltage (dV/dt) exceeded 5 mV/ms. The time of the first spike was measured from the start of the current step to the time of the first AP threshold. The amplitude was determined as the peak AP voltage relative to its threshold. The rise time was measured from 10% to 90% of the peak amplitude relative to the AP threshold. Half-width was determined as the AP width at the voltage level of its half amplitude relative to a threshold. The fAHP peak was determined as the point at which the voltage decay slowed to less than 5 mV/ms. The mAHP peak was measured as the lowest value of the voltage after the AP peak relative to its threshold. The time to mAHP was measured between the fAHP and mAHP peaks. ADP amplitude was measured as the peak voltage on the portion of the AP between the fAHP and mAHP peaks relative to the fAHP peak voltage.

For each neuron, the maximum AP frequency and the maximum slope of the current–frequency curves were measured. The current that elicited the maximum AP frequency was determined as the minimum current sufficient to elicit AP generation at the maximum frequency. Early frequency adaptation was determined as the ratio of the second interspike interval to the first, and late adaptation was determined as the ratio of the last interspike interval to the first at the next current step after the rheobase (rheobase + 10 pA).

### 4.4. Induction of Epileptiform Activity Ex Vivo

Epileptiform activity was induced in a brain slice by administering a proepileptic solution [61,76]. The solution consisted of 120 mM NaCl, 3.5 mM KCl, 1.25 mM NaH_2_PO_4_, 0.25 mM MgSO_4_, 2 mM CaCl_2_, 24 mM NaHCO_3_, 10 mM dextrose, and 0.1 mM 4-AP. LFP was recorded at least one hour after the induction of epileptiform activity.

### 4.5. Analysis of Epileptiform Activity

To automatically detect interictal and ictal discharges in LFP recordings, we first filtered the signal up to a sampling rate of 200 Hz using an 8th-order lowpass Butterworth filter. We chose the Butterworth filter because it preserves the shape of the AFC and avoids signal distortion in the passband. Next, we binarized the signal, with 1 representing the beginning of unitary epileptiform discharge and 0 representing the baseline. To determine the onset of uEEs, the threshold value was used. UEEs have amplitudes that are significantly greater than the average amplitude of the signal noise. This value was determined by analyzing the frequency histogram of signal amplitudes for each recording. According to the 4-AP model, the frequency of epileptiform discharge is approximately 1–2 Hz, so the threshold was set at the amplitude of the 99th percentile. The onset of uEEs was defined as the first time the signal amplitude exceeded the threshold. To avoid multiple determinations of uEEs within a single discharge, subsequent exceedances of the threshold within 50 ms within a single event were ignored. The proposed method accurately determines uEEs with a low false positive rate (less than 0.5%), as evaluated by experts. Identification of ictal discharges and the moments at which they occurred were determined by plotting the function of the cumulative number of interictal pulses. The spectrograms were obtained using a continuous wavelet transform. The Morlet wavelet was used because it is most appropriate for medical problems. The scales were chosen to resolve the entire frequency range of interest.

### 4.6. Histology

Rats were anesthetized with an aqueous solution of xylazine (10 mg/kg, Alfasan International B.V., Kuipersweg, The Netherlands) and zoletil (20 mg/kg, Virbac, Carros, France). Rats were transcardially perfused with 0.01 M phosphate-buffered saline (PBS, pH 7.4) followed by 4% paraformaldehyde (PFA) in PBS. Brains were removed and fixed in 4% PFA for 3–7 days at 4 °C. Finally, brains were cryoprotected in 30% sucrose solution and stored at −80 °C. Serial frontal sections, 20 µm thick and ranging from −2.6 to −3.6 mm from the bregma, were obtained using a Bright OTF5000 cryostat (Bright Instrument Co., Ltd., Huntingdon, UK).

Sections were Nissl stained as previously described [77]. Neuronal counts were performed using a Leica AF 7000 microscope (Leica Microsystems, Wetzlar, Germany). The number of neurons was counted per 100 μm for the cell layer in CA1 and CA3 (7–10 slices from a rat hippocampus) using ImageJ (U.S. National Institutes of Health, Bethesda, MD, USA).

Slices containing biocytin-filled pyramidal neurons were fixed with 4% PFA for 24 h at +4 °C. The slices were then rinsed in PBS for 3 × 10 min and incubated with 0.4% TritonX-100 (Merck, Darmstadt, Germany) in PBS for 2 h. Slices were then incubated with streptavidin conjugated to Alexa Fluor 488 (1:500 in PBS; #913909, Invitrogen, Waltham, MA, USA) for 24 h at +4 °C.

Neurons were reconstructed using a Leica TCS SP5 laser scanning confocal system (Leica Microsystems). Images were analyzed using the Leica Leica Application Suite Advanced Fluorescrence software package 2.3.6 build 5381 (Leica Microsystems) and ImageJ 1.53c software (U.S. National Institutes of Health, Bethesda, MD, USA). Dendritic spines on the apical dendrite of CA1 pyramidal cells were counted using the 2dSpAn software V.1 [78]. Only dendritic segments less than 5 µm in diameter were used for counting. The average density of spines per 1 µm of the apical dendrite located in the *str. radiatum* and *str. lacunosum moleculare*, respectively, was counted.

### 4.7. Statistical Analysis

Statistical data processing and graphing were performed using Statistica 8.0 (Systat Software, Inc., Palo Alto, CA, USA) and the OriginPro 8 program (OriginLab Corporation, Northampton, MA, USA). Outliers were identified using the Iglewicz and Hoaglin robust test for multiple outliers (two-tailed test). Normality of the sample data was assessed using the Kolmogorov–Smirnov test. The statistical significance of the difference between means was determined using an unpaired Student’s *t*-test for independent samples. Data are presented as mean and standard error of the mean.

## Figures and Tables

**Table 1:** Passive membrane properties of CA1 neurons in control and epileptic rats.

Membrane Property	Control (n = 27)	Epileptic (n = 40)	*p*
Resting membrane potential, mV	−62.6 ± 0.7	−63.4 ± 0.4	0.32
Input resistance, MΩ	82.5 ± 3.8	91.0 ± 4.0	0.15
Membrane τ, ms	19.9 ± 1.2	22.7 ± 1.5	0.17

**Table 2:** Properties of action potentials in CA1 neurons in control and epileptic rats.

Properties	Control (n = 27)	Epileptic (n = 40)	*p*
Hump amplitude, mV	0.95 ± 0.21	0.35 ± 0.11	0.10
Time of first spike, ms	521 ± 66	536 ± 55	0.87
Threshold, mV	−44.0 ± 0.6	−43.6 ± 0.3	0.47
Amplitude, mV	99.8 ± 0.8	97.8 ± 0.8	0.14
Rise time 10% to 90%, ms	0.34 ± 0.01	0.34 ± 0.01	0.84
Half-width, ms	1.28 ± 0.02	1.26 ± 0.02	0.35
fAHP, mV	−6.0 ± 0.4	−5.9 ± 0.5	0.95
mAHP, mV	−9.9 ± 0.3	−9.6 ± 0.3	0.48
Time to mAHP, ms	65 ± 3	74 ± 4	0.11
ADP, mV	1.9 ± 0.3	2.3 ± 0.3	0.38

## Data Availability

The data presented in this study are available upon request from the corresponding author.
